# Supplementary material for: Potential Inhibitors of Lumpy Skin Disease’s Viral Protein (DNA Polymerase): A Combination of Bioinformatics Approaches
Source: Animals (Basel). 2024 Apr 24;14(9):1283. doi: 10.3390/ani14091283 (PMC11083254; doi:10.3390/ani14091283)
Supplement: Supplementary file 1 [file animals-14-01283-s001.zip › Table S1.pdf]

Table S1: LSDV DNA replicating genes and their proteins.

Genes	Protein names	Functions	References
LSDV020	Ribonucleotide reductase, small subunit	DNA replication	UniProt Id.Q91MY6; [2]
LSDV039	DNA polymerase	DNA replication	UniProt Id.Q91MW8; [2]
LSDV045	DNA-binding phosphoprotein	DNA replication	UniProt Id.A0A1C9HI67; [2]
LSDV053	Glutaredoxin-2	DNA replication	UniProt Id.Q77GD7; [2]
LSDV066	Thymidine kinase	DNA replication	UniProt Id.Q77GD2; [2]
LSDV077	DNA topoisomerase	DNA replication	UniProt Id.Q77GC6; [2]
LSDV082	Uracil DNA glycosylase	DNA replication	UniProt Id.Q91MT0; [2]
LSDV083	NTPase	DNA replication	UniProt Id.Q91MS9; [2]
LSDV112	DNA polymerase processivity factor	DNA replication	UniProt Id.Q91MQ0; [2]
LSDV113	DNA polymerase processivity factor	DNA replication	UniProt Id.A0A8E7SPL8; [2]
LSDV133	DNA ligase	DNA replication	UniProt Id.Q91MN1; [2]
LSDV139	Ser/Thr protein kinase	DNA replication	UniProt Id.Q91MM5; [2]
